# Identification of a novel *FBN1* gene mutation in a large Pakistani family with Marfan syndrome

**Published:** 2012-07-18

**Authors:** Shazia Micheal, Muhammad Imran Khan, Farah Akhtar, Marjan M. Weiss, Farah Islam, Mehmood Ali, Raheel Qamar, Alessandra Maugeri, Anneke I. den Hollander

**Affiliations:** 1 Department of Biosciences, COMSATS Institute of Information Technology, Islamabad, Pakistan; 2 Department of Ophthalmology, Radboud University Nijmegen Medical Centre, Nijmegen, the Netherlands; 3 Department of Human Genetics, Radboud University Nijmegen Medical Centre, Nijmegen, the Netherlands; 4 Al-Shifa Eye Trust Hospital, Rawalpindi, Pakistan; 5 Department of Clinical Genetics, VU University Medical Center, Amsterdam, the Netherlands; 6 Shifa College of Medicine, Islamabad, Pakistan

## Abstract

**Purpose:**

To describe a novel mutation in the fibrillin-1 (*FBN1*) gene in a large Pakistani family with autosomal dominant Marfan syndrome (MFS).

**Methods:**

Blood samples were collected of 11 family members affected with Marfan syndrome, and DNA was isolated by phenol-extraction. The coding exons of *FBN1* were analyzed by polymerase chain reaction (PCR) and direct sequencing. One hundred-thirty controls were screened for a mutation in the *FBN1* gene that was identified in this family by restriction fragment length polymorphism (RFLP) analysis.

**Results:**

A novel heterozygous missense mutation c.2368T>A; p.Cys790Ser was observed in exon 19. This mutation substitutes a highly conserved cysteine residue by serine in a calcium binding epidermal growth factor-like domain (cbEGF) of FBN1. This mutation was present in all affected members and absent from unaffected individuals of the family in addition to 130 healthy Pakistani controls. Interestingly all affected family members presented with ectopia lentis, myopia and glaucoma, but lacked the cardinal cardiovascular features of MFS.

**Conclusions:**

This is a first report of a mutation in *FBN1* in MFS patients of Pakistani origin. The identification of a *FBN1* mutation in this family confirms the diagnosis of MFS patients and expands the worldwide spectrum of *FBN1* mutations.

## Introduction

Marfan syndrome (MFS) is an autosomal dominantly inherited syndrome with a prevalence of 1 in 5,000–10,000 individuals. The major clinical manifestations of the syndrome include three major systems according to the Ghent criteria i.e., the ocular, skeletal and cardiovascular systems [1,2].

Ocular features mainly involve ectopia lentis, which is observed in around 80% of MFS patients. Ectopia lentis is characterized by the dislocation of the lens, which typically occurs in patients between birth and 20 years of age after that lens is stabilized. Other features include high myopic eyes and retinal detachment in individuals aged 50–59 years [3,4]. According to the new Ghent criteria another cardinal feature of MFS is aortic root aneurysm/dissection. The most common physical features were craniofacial characteristics, high-arched palate, positive thumb and wrist signs. If the family history of the patient is not positive, the involvement of at least two organ systems is required to establish the diagnosis of MFS. Genetic screening of MFS can aid the diagnosis, as the presence of a mutation in the fibrillin-1 gene (*FBN1*) in the presence of a major manifestation of one organ system is sufficient to make the diagnosis [5].

There are three types of fibrillins in humans: FBN1, FBN2 and FBN3. Fibrillins are extracellular matrix fibrillar components which are essential for the correct function of elastic and nonelastic tissues including blood vessels, bone and eye [6]. Fibrillin-1 is a 350-kDa protein responsible for head-to-tail assembly of 10–12-nm fibrillin monomers in presence of calcium-constituting microfibrils. FBN1 forms a large multimeric protein complex by interacting with transforming growth factor beta (TGFβ), latent TGFb binding protein (LTBPs), and microfibrils that interact with bone morphogenetic protein (BMP) complexes. Depending on the requirements of a cell or tissue the FBN1 complex can activate extracellular matrix (ECM) sequestered growth factors or inhibit activated growth factors. Thereby defects in fibrillin and its associated structure e.g., due to mutation, potentially could activate the growth factor signaling pathway, that can lead to MFS and related disorders of the connective tissue [7].

FBN1 is composed of three types of repeated modules. The epidermal growth factor (EGF)-like modules contain six highly conserved cysteine residues, which form disulphide bonds with each other and are critical for the stabilized folding of the domain. FBN1 has 47 such modules, and 43 of them contain a calcium binding (cb) consensus sequence and are known as cbEGF-like modules [8]. The calcium ion bound in the cbEGF-like domain performs a crucial structural role in restricting the interdomain flexibility, which might have a role in protein–protein interaction [9]. The second type of module of FBN1 are transforming growth factor β1-binding (or TB) protein-like module (TGF β1-BP-like module, or 8- Cys/TB), which is found seven times in FBN1. This module contains eight cysteine residues that form four disulfide bonds. The third type of module is a hybrid module, which occurs twice [5].

Currently more than 1,200 mutations are known in the *FBN1* gene and missense mutations account for a major proportion (60%) of these mutations [10]. The majority of these mutations affect one of the cbEGF domains; often involving one of the six highly conserved cysteine residues within the cbEGF domains. Most mutations leading to a severe disease are found to be clustered in exons 24–32, which encodes a central stretch of 12 cbEGF repeats. This stretch is important in the formation of a rigid rod-like structure, which might be involved in the formation of microfibril assembly [11].

In this study we analyzed a five-generation family from Pakistan. All eleven affected family members lacked the cardinal cardiovascular features, but the diagnosis of MFS was confirmed by the identification of a novel mutation in the *FBN1* gene.

## Methods

### Patients and clinical data

In this study we recruited a five-generation consanguineous family with two loops, one from central Punjab and the other from the Azad Jamu and Kashmir area of Pakistan. Eleven out of 14 affected individuals and 5 of 6 normal healthy individuals participated in the study. 130 additional normal healthy controls were recruited for the study. After obtaining informed consent, thorough physical, ocular and cardiovascular examinations were performed for all participating family members.

### Molecular genetic analysis

Genomic DNA was extracted from whole blood using a conventional phenol-chloroform method [12]. PCR amplification of the 65 coding exons and flanking regions of the *FBN1* gene was performed in the proband (IV:13) using a PE 9700 thermocycler (Applied Biosystems, Foster City, CA). Primers used for PCR amplification are presented in Table 1. Briefly, for all amplicons the following cycling conditions were applied: initial denaturation at 94 °C for 5 min followed by 35 cycles of 94 °C for 30 s, 64 °C for 30 s, and 72 °C for 30 s. Sequencing reactions were performed using an ABI 3730 DNA analyzer from Applied Biosystems. For the detection of deletions and duplications, the SALSA MLPA kits P065 and P066 from MRC Holland (Amsterdam, the Netherlands) were used.

**Table 1:** Primer sequences of *FBN1*.

**Primer name**	**Forward primer sequence**	**Reverse primer sequence**	**T °C annealing**	**Product size**
FBN1-E01-M13-01F & 01R	CAAGAGGCGGCGGGAG	CGAACGGGGTGGGGACTAAACA	64	489
FBN1-E02-M13-01F & 01R	TATTTGGCCATCTCTTCCTCT	CCATGCAACCAACACAACA	64	240
FBN1-E03-M13-02F & 02R	TGGTCCCCTATAACAAATCGT	ATTGCAGGAAAGAGGAAAGC	64	288
FBN1-E04-M13-01F & 01R	AGCTGTTGCAATCTATGCATTTA	ATTCTACTTGTCTACAAACAGGT	64	246
FBN1-E05-M13-01F & 01R	CCACAAGTGTTACTTCATTAGCA	GTGCAAATTAGTAACAGCTTTAGG	64	260
FBN1-E06A-M13-01F & 01R	GCATGATGGTTCCTGCTT	AGACAATCCCGCTGAGTT	64	160
FBN1-E06B-M13-01F & 01R	CTGTGATCAGCAACCAGATG	GGCTCTCCAGAGCAAATAAG	64	262
FBN1-E07-M13-01F & 01R	CTGCAATGAATTTCATATGAGTTT	TTTTGCCTGCCCCCACTA	64	267
FBN1-E08-M13-01F & 01R	ACTGACGAATGGTTTTATATTGTG	AGTTGTTTGTTATGGAACTGACTT	64	279
FBN1-E09-M13-01F & 01R	GTTGTTACAAGTATTATCTCAGCG	GGCTGGGATGGGATATTCT	64	292
FBN1-E10-M13-01F & 01R	GCTACAGCTCAGCTGTTG	AATGTTAACTTGAACAATGCAAGA	64	310
FBN1-E11-M13-01F & 01R	CAACATCTTGTTCATTATTGTCAG	CAAGGAACAGAATTACAACAGAC	64	314
FBN1-E12-M13-01F & 01R	GGAACCCAGAAAGTCTTAGAATTA	GTTAGCATATATGTCCCACATTCC	64	270
FBN1-E13-M13-01F & 01R	ACTCCCCTAAATAAAGCTATTTCT	GCAATGGAAGGAGAGGACT	64	245
FBN1-E14-M13-01F & 01R	GCTTACTCTTCTGGTCATAAGAAA	AAAGGCACGTGAAGAACA	64	243
FBN1-E15-M13-01F & 01R	GCTGATGCTGCATATTATTTCCTA	TGAGTGACAGAGGCTGAAC	64	329
FBN1-E16-M13-01F & 01R	GGGTTCTCATCTGTTTGAAGT	CTCAATGGTGGCAGAAGG	64	294
FBN1-E17-M13-01F & 01R	CTGCAAACAAGGGAATCATT	TGATGCTGCCTCTGCACATA	64	182
FBN1-E18-M13-01F & 01R	TCCTGTAGCTCCTAAGGTCAT	ATTATGCAGGCAATGTTTCAGA	64	308
FBN1-E19-M13-01F & 01R	AGATACAGGCAAAGTTTGGG	CTAATGGCATTCCAAAAGATAGC	64	271
FBN1-E20-M13-01F & 01R	GGGTCAAAGTTGAAGTACTCT	GCAGGAAAAGCTGACATTAAG	64	296
FBN1-E21-M13-01F & 01R	ATTCCAAGGTGTATGTTTGAATTT	AGACCATTGGAGTGGTATAGG	64	278
FBN1-E22-M13-01F & 01R	ACTATGTCAGAACTGCAAAGTC	TATGACAGCTTTATCCAGTCCGA	64	243
FBN1-E23-M13-01F & 01R	ACTTACCAGGTTCAAAATGGG	CTAAGTGCTCAGCTATATCTTGT	64	348
FBN1-E24A-M13-01F & 01R	ACAGAGTGTTGGCAGTTTG	CTCCTCGTACTCAGGAGTATTT	64	268
FBN1-E24B-M13-01F & 01R	TGAGGAATGCGAGGAGTG	TGGGATGATCAAGTAGAGTGC	64	237
FBN1-E25-M13-01F & 01R	GCATTGAGACCTCCTGACT	GCCTTAATTCTTGCGACAATATG	64	307
FBN1-E26-M13-01F & 01R	AAGGCTGTCCTGAGACTC	GCTTCATGGAATCCTTCTCTT	64	259
FBN1-E27-M13-01F & 01R	TGGTGGAGGAGATGAGGC	GCAATGATGTCATTCAAACAACTG	64	270
FBN1-E28-M13-01F & 01R	TTCACACCATTTACTTGTGGTC	ACATAGAGTGTTTTAGGGAGAGAT	64	367
FBN1-E29-M13-01F & 01R	ACGAGTATTGGAGGGGAC	TAGGAACCTACTGAGAGATTCAAC	64	266
FBN1-E30-M13-01F & 01R	ACTGAACAGTGGAACCAATATCAA	TGCTTATGACTAACAAGACAAGAT	64	263
FBN1-E31A-M13-01F & 01R	GTCATAGTTATTATGTCTCGAGGG	GTGGCAGATAAATGAGCCTT	64	193
FBN1-E31B-M13-01F & 01R	ATCTGCCTAAGTGGGACC	ACAAATTTCAAAGAAGTGGAAGC	64	291
FBN1-E32-M13-01F & 01R	GACATTTGTGCTGAGCCT	ATGTGTAATCTATGCAGTCCTTG	64	254
FBN1-E33-M13-01F & 01R	ATTGGTTTTAAATACCACCCTTTC	TGGCTTCTCTGACTAGTGTTG	64	258
FBN1-E34-M13-01F & 01R	CTAACCGAGGAAGAGTAACG	TCTCATCAAGCCCAGCAAG	64	245
FBN1-E35-M13-01F & 01R	GATTGGTGTTAGATACTCTGCATT	GACACCAGGGAGCTGATT	64	315
FBN1-E36-M13-01F & 01R	CCTACACTGGCTCAGGTGATAA	ACACAGTATGCTTGCTTCTC	64	245
FBN1-E37-M13-01F & 01R	AGAAAGATTCTGCCTGATGC	GAGAACTGGCTGGAGTTGA	64	292
FBN1-E38-M13-02F & 02R	ACAAAGGTGTTAACTTACTTCAGAC	TAAACCCAAGGAAATTCAAGTTGTG	64	311
FBN1-E39-M13-01F & 01R	TTCCTTGGGTTTATTTACAATGCT	CAGGTCAGTTCTTGATATCTGC	64	265
FBN1-E40-M13-01F & 01R	GGCCATTCCAAAATGTGAAG	GATGAGAACCAAACATGCATTAC	64	256
FBN1-E41-M13-01F & 01R	GTGATTTCCCACATGGCA	TTTCCCCAACAATTCATGGGTAA	64	314
FBN1-E42-M13-01F & 01R	TCCAATTATTGTTCTTTGCTGACC	AAATAATGCTAACACAAAGGCAAA	64	225
FBN1-E43-M13-01F & 01R	TGTGCTGTCCTGTCACTC	GTAGCTCATCAGTTAGCTCTTT	64	253
FBN1-E44-M13-01F & 01R	GTTGACTGGACACCAGATT	GAAGACAAACTCTTGGGTAGG	64	260
FBN1-E45-M13-01F & 01R	CTCCTGAGAATGATAGCTAGAAGT	CAAATGAAGCTTTCAACAGCATA	64	294
FBN1-E46-M13-01F & 01R	CCGTGTAACCACTTTTTCTACT	CTGGAACACTAGAGATGATGCTAA	64	291
FBN1-E47-M13-01F & 01R	TTGATTATTGCTGGGATTATGACA	ATGATTCCTTGAGTGGTCTCT	64	288
FBN1-E48-M13-01F & 01R	CAGTGGGAACCTCTTCCTTA	CCGACACTCCTCATTTGCT	64	231
FBN1-E49-M13-01F & 01R	CTGATGATGTCTCCATCGTG	TGCAGCATTGAAAGCCCA	64	250
FBN1-E50-M13-01F & 01R	TGCAATACGGACTCAGTAGG	TACTTACATCATGGCCAGTCT	64	302
FBN1-E51-M13-02F & 02R	AGCATGTAGCAATTTTCTACCT	AAGAATAACTAGAGAAGAAGCAGAT	64	259
FBN1-E52-M13-01F & 01R	CTTCACGTTTAAAAAATACCTTGT	AGTGCCATCTTGGTACCTAT	64	301
FBN1-E53-M13-01F & 01R	ACAACAACAACAACAAAATTACAG	TGTTCCCAGGATCAGTACAC	64	303
FBN1-E54-M13-01F & 01R	TTGCTGTCCATGATCCCT	TTGCTGTCCATGATCCCT	64	245
FBN1-E55-M13-01F & 01R	AAAGTCAGGTAATTAAGGCAGATA	CTTCTGATGCACTCAAAGCTC	64	274
FBN1-E56-M13-01F & 01R	AATGGTCAGATGACTCTTCTTG	GTGTGGAGGCTGAGGTTAG	64	253
FBN1-E57-M13-01F & 01R	TGCTCTTAAAATTTCCTGACATCC	ACAAATAAATAGATTCCCTGCAAG	64	338
FBN1-E58-M13-01F & 01R	AGTATTTACACTGAAGTGACCC	AAAATTTCCACTTGAGGATAAGC	64	285
FBN1-E59-M13-01F & 01R	TGAGCGTGTACACATCATTT	GGAATGCAGCCATGTGTC	64	268
FBN1-E60-M13-01F & 01R	CCTGTTTTGTTGGCTTGAC	GAATCGCTACAATCCATGTAGG	64	240
FBN1-E61-M13-01F & 01R	ATGATACAAAGAGAGCTTTGGG	CCTCCACAAGGATTCACCA	64	269
FBN1-E62-M13-01F & 01R	CTTCAGAGAGAGATGTTGAGTTG	TGTTTTGCTTCATAGGACCTGATA	64	263
FBN1-E63A-M13-01F & 01R	TCAATAGAAATCTCTGGCTGCT	TCCTCCACTGAACTGTTCATAC	64	202
FBN1-E63B-M13-01F & 01R	TACAAGTGCATGTGTCCCG	ACGAATGAAAGAATCTCCAACC	64	245
FBN1-E64-M13-01F & 01R	CCTACCTTGTCTTCCCATTCTAA	TTCCACCACAGGAGACAT	64	305
FBN1-E65A-M13-01F & 01R	CTTTAATATGAGAGCTAAGTGGCA	AGCCATCTTCATTTCCAGATTC	64	278
FBN1-E65B-M13-01F & 01R	CTCTGACGAATCACAACAGATAC	ATATGATGATTCTGATTGGGGGA	64	377

Segregation of a novel missense change in exon 19 was performed by direct sequencing of this exon in other family members using standard conditions. The forward primer 5′-CAG GAG TTT TGC CTT TTT GC-3′ and reverse primer 5′-TGC CAT GTA GAA CCA CAG AA-3′ were used to amplify a 394 base pair (bp) product containing exon 19. PCR products were visualized on 2% agarose gel and purified by using PCR clean-up purification plates (NucleoFast® 96 PCR; MACHEREY-NAGEL, Düren, Germany), according to the manufacturer’s protocol. Purified PCR products were analyzed by Sanger sequencing in an automated DNA sequencer (Big Dye Terminator, version 3 on a 3730 DNA analyzer; Applied Biosystems). Sequencing results were assembled and analyzed by using Vector NTI Advance™ 2011 software from Life echnologies/Invitrogen (Bleiswijk, The Netherlands).

Unrelated control individuals were analyzed for the novel mutation by restriction fragment length polymorphism (RFLP) analysis using the restriction enzyme AlwNI (New England Biolabs, Ipswich, MA). PCR products were digested by using 2 U of enzyme, 1× PCR buffer 4 (20 mM Tris-acetate, 50 mM potassium acetate, 10 mM Magnesium Acetate, 1mM Dithiothreitol pH 7.9) and 16 µl of amplified PCR product, and incubated for 2 h at 37 °C. After heat inactivation at 65 °C the product was separated on a 3% agarose gel.

## Results

### Clinical characteristics

**Figure 1:**
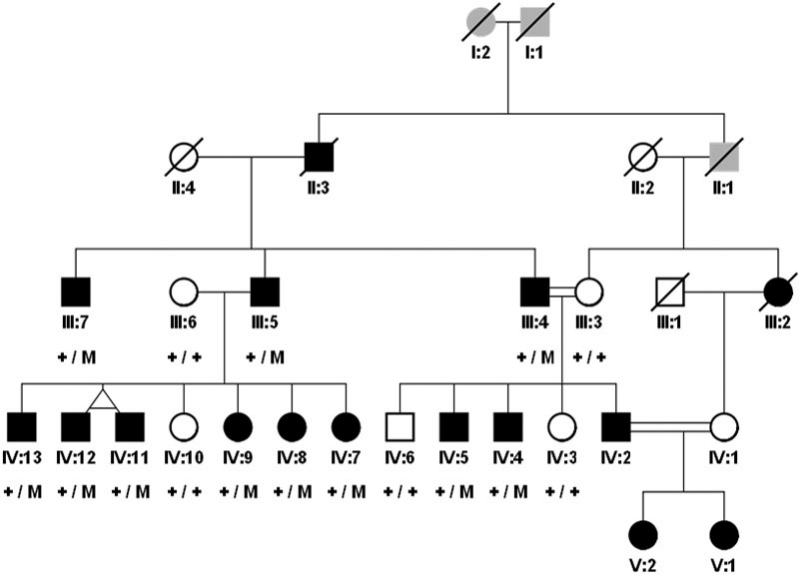
Pedigree of a Pakistani family with MFS. Squares indicates males and circles females, black symbols represents affected and white unaffected individuals, gray symbols indicates unknown affection status. Slashed symbols represent the deceased subjects. + indicates the normal allele, and M indicates the Cys790Ser mutation.

Eleven affected individuals of the family including 8 males and 3 females participated in the study (Figure 1). All patients showed similar clinical symptoms. In all affected members bilateral lens dislocation occurred and lenstectomy was performed. Other ocular symptoms included high myopia and glaucoma. None of them displayed any cardiovascular system abnormalities on echocardiography. Abnormalities of the skeletal system in MFS such as tall stature, long limbs, joint hypermobility, long narrow head, arachnodactyly, flat feet and medial displacement of medial malleoli, hollow cheeks and recessed or protruding jaw were noted in all individuals (Table 2).

**Table 2:** Clinical evaluation of affected family members.

**Patient ID**	**Manifestation**	**III:4**	**III:5**	**III:7**	**IV:4**	**IV:5**	**IV:7**	**IV:8**	**IV:9**	**IV:11**	**IV:12**	**IV:13**
Age (years)		55	40	35	30	28	12	16	8	20	6	6
Gender		M	M	M	M	M	F	F	F	M	M	M
**Ocular System**												
(i)	Ectopia lentis	+	+	+	+	+	+	+	+	+	+	+
(ii)	Myopia	+	+	+	+	+	+	+	+	+	+	+
(iii)	Abnormally flat cornea	-	-	-	-	-	-	-	-	-	-	-
(iv)	Early development of nuclear cataract	+	+	+	-	-	-	+	-	+	+	+
(V)	Glaucoma (IOP)	R34	15	R14	R15	R14	R20	R16	R12	R18	R14	R18
		L34	15	L14	L12	L16	L24	L16	L14	R16	L12	L20
(Vi)	Retinal detachment	+	-	+	+	-	-	-	-	-	-	-
**Cardiovascular system**												
(i)	Aortic root dimension (mm)*	32	31.8	23.6	35.1	27.5	25.1	28.7	20	34	35	20
(ii)	Mitral valve prolapse	-	-	-	-	-	-	-	-	-	-	-
(iii)	Dilation of pulmonary artery	-	-	-	-	-	-	-	-	-	-	-
**Skeletal system**												
(i)	Height (H; cm)	176.7	173.4	167.6	170.6	167.4	146.3	158.5	121	167.6	106.6	106.6
(ii)	Arm span (AS; cm)	182	177	172	175	173	153	164	125	175	110	110
(iii)	AS/H (normal <1.05)	1.03	1.02	1.02	1.02	1.03	1.04	1.03	1.03	1.04	1.03	1.03
(iv)	Pectus carinatum	-	-	-	-	-	-	-	-	-	-	-
(v)	Pectus excavatum	-	-	-	-	-	-	-	-	-	-	-
(vi)	Arachnodactyly	+	+	+	+	+	+	+	+	+	+	+
(vii)	High palate with dental crowding	+	+	+	+	+	+	+	+	+	+	+
(viii)	Flatfoot	+	+	+	+	+	+	+	+	+	+	+
(ix)	Characteristic face (long narrow head, hollow cheeks, recessed or protruding jaw)	+	+	+	+	+	+	+	+	+	+	+
**Other manifestations**												
(i)	Hyperextensible skin	-	-	-	-	-	-	-	-	-	-	-
(ii)	Striae	-	-	-	-	-	-	-	-	-	-	-
(iii)	Hernia	+	+	+	+	+	-	-	+	+	-	-

IOP: intraocular pressure, R: right eye, L: left eye. *normal range 20–37 mm.

### Identification of a novel *FBN1 mutation*

**Figure 2:**
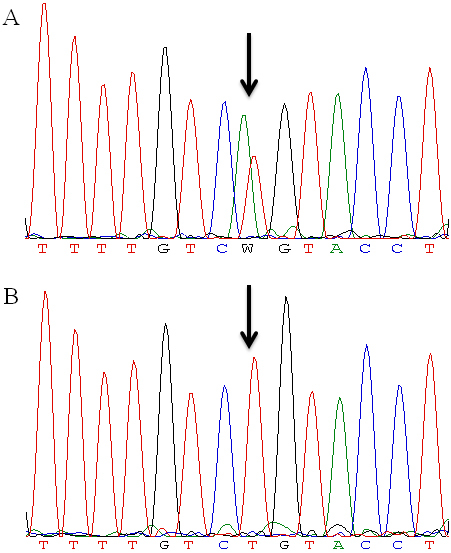
Sequence traces of the novel FBN1 missense mutation Cys790Ser in exon 19. **A**: A heterozygous change T>A (indicated by arrow) is identified in an affected family (IV:13) member. **B**: The corresponding normal sequence in an unaffected family member (IV:10).

Direct sequencing of *FBN1* revealed a novel heterozygous mutation (c.2368T>A) in exon 19 which results in a change from a cysteine to a serine (p.Cys790Ser; Figure 2A,B). This missense mutation was present heterozygously in all affected members of the family while it was absent from normal individuals of the family as well as 130 unrelated healthy controls.

**Figure 3:**
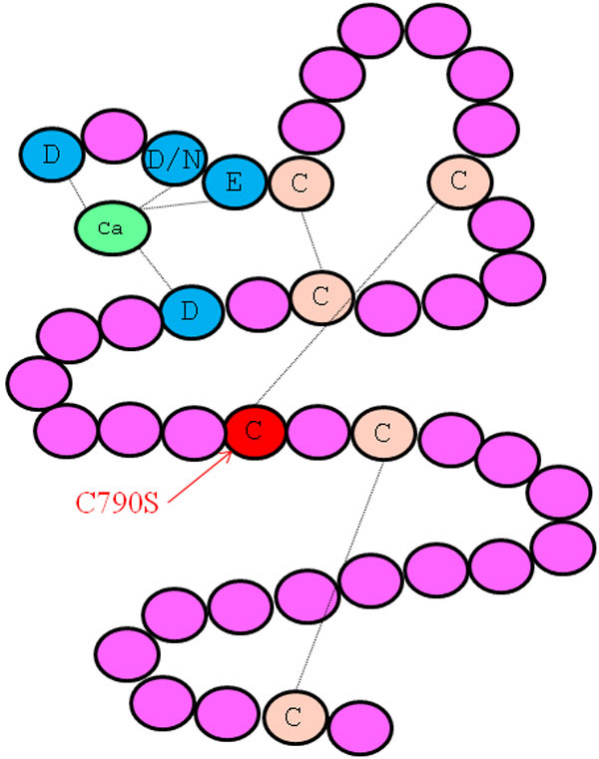
A schematic representation of the consensus secondary structure of a prototypical cbEGF-like domain. Calcium binding in the NH_2_-terminal region of the wild-type domain is mediated by the highly conserved amino acids highlighted in blue. The highly conserved cysteines of the cbEGF-like domain are marked with a C, and the lines between the cysteine residues represent disulfide bridges. The mutation Cys790Ser affects the 4th cysteine residue, which is predicted to form a disulfide bridge with the 2nd cysteine residue (Cys776). The mutation abolishes the disulfide bond formation and thus causes misfolding of the protein.

The p.Cys790Ser mutation resides in the 12th cbEGF domain, where it affects one of the highly conserved cysteine residues. Cys790 is the 4th cysteine residue of this cbEGF domain, which is predicted to form a disulphide bond with the 2nd cysteine residue (Cys776; Figure 3).

## Discussion

Of all mutations that have globally been identified in the *FBN1* gene, 38.6% result in a truncated FBN1 protein, and 60.3% represent missense mutations and of which most (78%) are localized in the cbEGF-like modules [13]. In the 43 cbEGF domains each have six highly conserved cysteine residues (C1-C6), which form disulphide bonds among each other (C1-C3, C2-C4, C5-C6). We identified a novel mutation in exon 19 of the *FBN1* gene in a large MFS family from Pakistan, which, is predicted to abolish the C2-C4 (Cys776-Cys790) disulphide bond of the 12th cbEGF domain, as the 4th cysteine residue is replaced with serine (Cys790Ser). Missense mutations in *FBN1* that affect the cysteines, which are essential for the correct EGF-like domain structure, act in a dominant negative manner. Since the monomers from the mutated allele are folded incorrectly, they assemble with the normal monomers from the other allele creating abnormal multimers [14,15].

So far, no clear genotype-phenotype correlations in *FBN1* have yet been established, though some correlations have been suggested in some studies, which included a large number of individuals (n=101, 93, 57, 81, and 76 patients). Interestingly, a higher frequency of cysteine substitutions was observed in MFS patients with ectopia lentis, opposed to premature termination codon mutations [16-20]. This is in line with the clinical findings in the family described in this study, as all affected family members developed ectopia lentis.

Previously most of the *FBN1* mutations were found in other exons rather than exon 19. To date only four mutations have been identified in exon 19: a frameshift mutation observed in one Italian patient with clinical symptoms mainly involving the skeletal and cardiovascular systems [21], and three missense mutations in 3 sporadic patients from Belgium with classical MFS and involvement of the cardiovascular system [1]. All three missense mutations were present in the 8th cbEGF domain affecting the 2nd and 3rd cysteine residues. Though all mutations identified in exon 19 have so far been associated with involvement of the cardiovascular system, this is not the case in the family described in this study, as none of the affected family members showed cardiovascular system abnormalities on echocardiography.

It has been reported that mutations in exons 24–32 are found in MFS individuals with a more severe and complete phenotype, including a younger age at diagnosis and a higher probability of developing ectopia lentis, ascending aortic dilatation, aortic surgery, mitral valve abnormalities, scoliosis, and shorter survival [11]. The ocular manifestations in this family, carrying a mutation in exon 19 of *FBN1*, were severe. All patients, including 4 young children, developed ectopia lentis, myopia and glaucoma. However, no cardiovascular system defects have been developed in the affected individuals so far. Moreover, among skeletal manifestations no pectus abnormalities or scoliosis have been noted.

Loeys et al. [1] did not find distinguishing features in patients with MFS with or without a *FBN1* mutation except for the presence of ectopia lentis, which was significantly higher in individuals with mutations in *FBN1*. A significant difference was observed between the clinical phenotype of patients carrying a missense mutation in 11th and 12th EGF-like domains compared with patients carrying a missense mutation in EGF-like domains 13–18. Patients with mutations in EGF-like domains 11 and 12 have a shorter survival, a younger age at diagnosis, a higher probability of neonatal presentation, and a higher risk of developing ascending aortic dilatation than patients with a missense mutation affecting EGF-like domains 13–17 [11]. In the Pakistani family, carrying a mutation in the 12th EGF-like domain, patients were also diagnosed at a young age and some of them have a neonatal presentation, but no cardiovascular symptoms were observed.

Marfan syndrome is characterized by a high clinical heterogeneity. The presentation of cardinal symptoms varies among families but also within families. The prevalence of cardiovascular features such as mitral valve prolapse is 43% and ascending aortic dilation is 53% in individuals in the age group <30. It increases steadily with age to 75% and 96%, respectively, in individuals of ≤60 years with *FBN1* mutations [22]. The affected individuals of the 5-generation family we present here lack the cardinal cardiovascular features of MFS, but do have the typical skeletal and ocular symptoms. The novel mutation in the *FBN1* gene identified in this family supports the diagnosis of MFS.

Although cardiovascular features are absent in the family, it has been reported that in some cases these features may not manifest until adulthood. Black et al. [23] reported the development of aortic root dilatation in the fifth decade of life in patients presenting familial ectopia lentis at the age of 58 and 70 years. In the present family only one affected individual has reached age 50, whereas three are in the age group of 30–40 years, four affected individuals are in their childhood and 3 are infants. Therefore the young age might explain the absence of cardiovascular manifestation in these individuals. The study of Black et al. [23] confirms the need for life-long screening in adult patients carrying a *FBN1* mutation in the absence of major manifestations.

In this family of Pakistani origin affected individuals experience major involvement of the ocular and skeletal systems. Interestingly, all patients in this family developed ectopia lentis, had high myopia and glaucoma. In literature it has been reported that glaucoma occurs in 64% of patients with MFS and in 10% of patients with isolated ectopia lentis [24]. Glaucoma has been observed more frequently in spontaneous late subluxation of the lens than in the congenital type. However, in the family described here glaucoma was been observed in combination with early sublaxation of the lens in all affected individuals, suggesting that predisposing factors may be present in this family, adding to the risk of developing glaucoma.

To the best of our knowledge this is the first report of a family with *FBN1* mutations, in which all affected individuals developed glaucoma and myopia. The presence of these particular clinical features in all affected individuals might be due to the novel mutation p.Cys790Ser, or alternatively may be associated with additional genetic factors that contribute to the disease phenotype in this family. We believe that the p.Cys790Ser mutation affects the structure of the protein by disturbing the arrangement of the microfilaments, and acts in a dominant negative manner. Although more than 1,200 mutations in the *FBN1* gene have been identified for MFS, this is a first report from Pakistan, which expands the worldwide mutation spectrum and adds to the existing knowledge of genotype-phenotype comparisons for MFS.
